# Is elevated SUA associated with a worse outcome in young Chinese patients with acute cerebral ischemic stroke?

**DOI:** 10.1186/1471-2377-10-82

**Published:** 2010-09-18

**Authors:** Bin Zhang, Cong Gao, Ning Yang, WeiZhi Zhang, XingWang Song, JianRui Yin, ShuXiang Pu, YongHong Yi, QingChun Gao

**Affiliations:** 1 Key Laboratory of Neurogenetics and Channelopathies of Guangdong Province and The Ministry of Education of China, Institute of Neuroscience and The Second Affiliated Hospital of GuangZhou Medical University, 250# Changgang east Road, GuangZhou, 510260 Guangdong Province, China; 2 Department of Neurology, the Second Affiliated Hospital of GuangZhou Medical University, 250# Changgang east Road, GuangZhou, 510260 Guangdong Province, China; 3 Department of Urinary Surgery, the First Affiliated Hospital of DaLian Medical University, 222# Zhongshan Road, DaLian, 116011 LiaoNing Province, China; 4 Gangwan Hospital of Guangzhou Medical University, 621# Gangwan Road, GuangZhou, 510700 Guangdong Province, China

## Abstract

**Background:**

Elevated serum uric acid (SUA) levels can enhance its antioxidant prosperities and reduce the occurrence of cerebral infarction. Significantly elevated SUA levels have been associated with a better prognosis in patients with cerebral infarction; however, the results from some studies on the relationship between SUA and the prognosis of patients with cerebral infarction remain controversial.

**Methods:**

We analyzed the relationship between SUA and clinical prognosis of 585 young Chinese adults with acute ischemic stroke as determined by the modified Rankin Scale at discharge. Using multivariate logistic regression modeling, we explore the relationship between SUA levels and patient's clinical prognosis.

**Results:**

Lower SUA levels at time of admission were observed more frequently in the lowest quintile for patients with severe stroke (P = 0.02). Patients with cerebral infarction patients caused by small-vessel blockage had higher SUA concentrations (P = 0.01) and the lower mRS scores (P < 0.01) were observed in, while the lowest SUA concentrations and the highest mRS scores were seen in patients with cardiogenic cerebral infarction patients. Logistic regression analysis adjusted for confounders confirmed the following independent predictors for young cerebral infarction: uric acid (-0.003: 95%CI 0.994 to 0.999) and platelet (0.004, 95%CI 0.993 to 0.996).

**Conclusion:**

Elevated SUA is an independent predictor for good clinical outcome of acute cerebral infarction among young adults.

## Background

Stroke in young adults is an important cause of lifelong disability. Thus it is very important to study the pathogenesis and prognosis of young patients with cerebral infarction. Conventional risk factors for arterial thrombosis at young age, such as gender, smoking, diabetes mellitus, obesity and hypercholesterolemia do not fully explain the cerebrovascular risk, Therefore additional factors may exist which contribute to the likelihood of developing arterial thrombosis in young adults. Studies have shown that elevated SUA can increase the risk of hypertension, diabetes, dyslipidemia, obesity, and renal failure [1-3].

More recent researchers have established a relationship between elevated SUA and vascular events (mainly cardiovascular disease), and it is believed that elevated serum uric acid levels could increase the morbidity and mortality from cerebral infarction [4-7]. However, other large-scale studies have found that SUA is a powerful free radical scavenger in humans, and SUA concentration in normal adults is almost 10-folder higher than that of other antioxidants [8]. Cerebral infarction initiates a complicated cascade of metabolic events and produce lots of free-radical that mediated oxidative damage in the central nervous system [9].

Studies with experimental cerebral ischemia have demonstrated the neuroprotective effects of SUA. SUA could scavenge superoxide, hydroxyl radical, oxygen, and chelate transition metals. Beside its ability to react with potent oxidants, SUA is also able to almost completely prevent the inactivation of SOD3 by H2O2 [10,11]. Clinical studies with confounders well-controlled demonstrated that higher levels of SUA in stroke patients on admission predicted better prognosis [12].

These contradictory findings may be related to experimental designs and condition definitions. In other words, could higher levels of SUA at admission predict better outcomes in stroke patients? We try to answer this question in a large series of young patients with acute ischemic stroke.

## Methods

All consecutive patients between the ages of 18 and 45 years with first-ever cerebral infarction during 2001-2009 were recruited to participate in the study. The Second Affiliated Hospital of GuangZhou Medical University is the largest city hospital, covering about 250 million people, was the main center for the study. Another group of patients from the Second Affiliated Hospital of Guangzhou Medicine University, Guangzhou, in China were consecutively enrolled in the study. All patients were Chinese. All patients were admitted within 48 hours of experiencing a new focal or global neurological event. Brain imaging (either CT or MRI) was performed routinely within 24 to 48 hours after admission. We excluded patients with cerebral infarction from other intracranial-related diseases such as subarachnoid hemorrhage, sinus venous thrombosis, or severe head trauma.

Cerebral infarctions were classified according to the TOAST system [13]. The modified Rankin Scale (mRS) was available and utilized in 585 patients. The mRS score at discharge was classified as independent (score 0 to 2) or dependent/dead (score 3 to 6).

At baseline, demographic data (age and sex) and history of conventional vascular risk factors (hypertension, diabetes mellitus, atrial fibrillation, hyperlipidemia, smoking habit, alcohol abuse) were obtained. Routine blood and biochemical tests, ECG, and a baseline brain CT/MRI scan were performed in all patients at admission. Laboratory investigations for vascular risk factors, duplex sonography of the carotid and vertebral arteries, and a thorough cardiac investigation were all taken. All patients received treatment according to current guidelines, but none of them underwent thrombolysis or surgical treatment.

All patients had given informed consent to participate in the study. Permission for the study was obtained from the local ethics committees. The 330 healthy patient control groups come from hospital staffs, and exclusion factors in the control group included a history of stroke, dementia, Parkinson's disease, multiple sclerosis, renal failure, severe traumatic brain injury, brain tumor and other diseases affecting the SUA level. These people had never taken drugs that could potentially confound SUA concentration.

### UA measurement

All blood samples were collected on the first day of admission under fasting state, and SUA was measured in accordance with standard detection methods in the hospital biochemistry department of this hospital. The specific method: Non-fasting blood was collected and centrifuged. Within 30 minutes, the blood was centrifuged for 10 minutes at 3000 rotations per minute at room temperature. Subsequently, SUA activity was determined with a Kone Diagnostica reagent kit and a Kone autoanalyzer. External quality control program showed that the interassay coefficient of variation was lower than 4.1%.

### Statistical Analyses

Statistical analyses were performed with the χ^2 ^test for binary and categorical data and the Mann-Whitney U test for continuous variables. The severity of the cerebral infarction was classified as mild (mRS score 0 to 2: absent or mild neurological symptoms) and moderate/severe (mRS score 3 to 6: moderate or severe disability/death) according to the mRS score at discharge [14].

Multivariate analysis was performed by binary logistic regression analysis, which allows adjustment for confounding factors. All variables have an inclusion criterion of P < 0.10 that were at least weakly associated with stroke severity. In the binary logistic regression model, the history of TIA/stroke, diabetes mellitus, atrial fibrillation, serum fibrinogen, hypertriglyceridemia, hypercholesterolemia, TOAST type, serum triglyceride and cholesterol levels, serum platelet and PDW levels, serum leukocyte, SUA and glucose levels were used to examine the effect of these variables in the prediction of group status (mRS score 0 to 2, mRS score 3 to 6). The analyses were undertaken with the SPSS (version 11.1) software package.

## Results

### Clinical Characteristics

A total of 426 young patients had better prognosis at discharge (mRS 0-2), while 159 young patients who had a worse prognosis (mRS 3-6). As shown in Table 1, those patients with poor outcomes were more likely to have risk factors such as diabetes, hypertriglyceridemia, metabolic syndrome presence, moyamoya/cerebral vascular malformation more frequently. furthermore, they rarely suffered from history of stroke/TIA.

**Table 1:** Baseline Characteristics of Study Population in Relation to Stroke Severity According to mRS Score at discharge

Characteristic	MRS 0-2 (n = 426)	MRS 3-6 (n = 159)	*P*
Male/Female	283/143	107/52	0.844
Age, y	39.6 ± 6.5	39.2 ± 6.5	0.504
Current cigarette smoking, n (%)	120 (28.2%)	48 (30.2%)	0.631
Current Alcohol drinking, n (%)	78 (18.3%)	30 (18.9%)	0.877
Previous stroke/TIA, n (%)	56 (13.1%)	9 (5.7%)	0.010
Atrial fibrillation, n (%)	14 (3.3%)	24 (15.1%)	0.000
Hypertention, n (%)	190 (44.6%)	76 (47.8%)	0.49
Diabetes at baseline, n (%)	70 (16.4%)	43 (27.0%)	0.004
Hypercholesterolemia, n (%)	137 (32.2%)	41 (257%)	0.136
Hypertriglyceridemia, n (%)	161 (37.8%)	43 (27.0%)	0.015
Metabolic syndrome presence, n (%)	21 (4.9%)	10 (6.3%)	0.003
Family history of stroke, n (%)	19 (4.5%)	13 (8.2%)	0.079
Moyamoya/Cerebral vascular malformation, n (%)	14 (3.3%)	13 (8.2%)	0.012
TOAST classification			0.000
a. Large artery, n (%)	127 (29.8%)	84 (52.8%)	
b. Small artery, n (%)	226 (53.1%)	19 (12.0%)	
c. Cardioembolism, n (%)	21 (4.9%)	31 (19.5%)	
d. Other cause, n (%)	52 (12.2%)	25 (15.7%)	
nonfasting glucose, mmol/L	5.65 ± 2.44	6.44 ± 3.03	0.001
Platelet distribution width, fL	12.7 ± 4.6	12.1 ± 3.0	0.072
Platelet count, **×**10^9^/L	237.8 ± 80.0	224.9 ± 82.9	0.019
White cell count, **×**10^9^/L	8.6 ± 5.6	10.0 ± 4.2	0.000
Mean platelet volume, fl	10.2 ± 1.4	10.2 ± 1.5	0.85
UA, mmol/L	326.2 ± 103.4	295.7 ± 111.2	0.002
Triglyceride (TG), mmol/L	1.7 ± 1.4	1.6 ± 1.6	0.382
Cholesterol (CHOL), mmol/L	4.7 ± 1.1	4.9 ± 1.1	0.017
Fibrinogen, g/l	1.7 ± 0.4	2.7 ± 1.7	0.000

Blood tests revealed that in those patients with worse prognosis, the number of white blood cells, serum glucose, serum CHOL, serum fibrinogen levels on admission significantly increased, in contrast, SUA and platelet count significantly decreased.

The admission SUA levels were usually in the lowest quintile for patients with worse prognosis (P = 0.02, Table 2).

**Table 2:** Association Between UA and Stroke Severity According to mRS Score at discharge

UA Quintiles	mRS 0-2 n = 426	mRS 3-6 n = 159	P
1 (76-227 mmol/L)	73 (17%)	45(28%)	0.019
2 (228-285 mmol/L)	90(21%)	29(18%)	
3 (286-336 mmol/L)	80(19%)	34(22%)	
4 (337-403 mmol/L)	91(21%)	29(18%)	
5(404-673 mmol/L)	92(22%)	22(13%)	

Cut points for quintiles are 227 mmol/L, 285 mmol/L, 336 mmol/L,403 mmol/L.

The relationship between Admission SUA Concentration and Demographics and Risk Factors

As shown in table 3, there were significantly increased SUA concentration in male patients with hypertension, current smokers, hypertriglyceridemia and hypercholesterolemia.

**Table 3:** Uric Acid Concentration on Admission in different clinical Demographics

Variable	yes	No	*P*
Female sex	273.0 ± 96.0	340.3 ± 104.2	0.000
Hypertension	334.7 ± 110.9	304.0 ± 100.4	0.001
Current smoking	346.4 ± 95.7	306.5 ± 108.3	0.000
Current Alcohol drinking	313.6 ± 113.4	318.5 ± 103.1	0.660
diabetes	326.5 ± 106.0	317.1 ± 106.5	0.561
Prior TIA/stroke	320.0 ± 97.7	317.5 ± 107.8	0.638
Atrial fibrillation	321 ± 132.7	317.5 ± 105	0.767
Hypertriglyceridemia	343.6 ± 106.3	306.8 ± 105.3	0.000
Hypercholesterolemia	357.6 ± 102.6	297.9 ± 103.5	0.000

In table 4, patients with cerebral infarction caused by small artery occlusion had the highest SUA concentrations and the lowest mRS score. In contrast, patients with cerebral infarction from cardiogenic causes had the lower SUA concentrations and the highest mRS scores.

**Table 4:** UA concentrations in different stroke subtype

TOAST	All ptients n = 585	mRS
a. Large artery	319.5 ± 108.5	2.1 ± 1.4
b. Small artery	333.6 ± 98.3	0.9 ± 0.9
c. Cardioembolism	313.6 ± 133.4	2.7 ± 1.7
d. Other cause	293.8 ± 106.2	2.0 ± 1.6
P	0.01	0.000

Uric Acid Concentration on Admission in Relation to clinical prognosis

As shown in table 5, stepwise multiple logistic regression analysis confirmed that the independent positive relation between higher SUA levels on admission and better prognosis at discharge. Moreover, higher serum platelet levels on admission were also associated with better prognosis (table 5). We also found a correlation between higher serum fibrinogen and leukocyte, the worse outcome. Meanwhile, diabetes is an independent risk factor for young patients with cerebral infarction.

**Table 5:** Independent Predictors of Functional Outcome at Hospital Discharge

	OR (95% CI)	*β*	P	OR
UA	0.994-0.999	-0.003	0.006	0.997
PLT	0.993-0.999	-0.004	0.020	0.996
Fibrillation	1.691-11.389	1.479	0.002	4.389
Diabetes	1.193-3.674	0.739	0.010	2.094
Leukocyte	1.078-1.257	0.152	0.000	1.164

We have tried to determine a possible relationship between SUA and patients prognosis 3 months after cerebral infarction. However, the mRS after 3 months was available in only 252 of the 585 patients (43%).

Finally, we compared the SUA levels in the control healthy people and the cerebral infarction patients (Table 6). The results showed that SUA levels in the normal group were much higher than in young patients with cerebral infarction (P = 0.023).

**Table 6:** UA concentrations in normal control and all patients

	Sex(M/F)	Age (years)	UA (mmol/l)
Healthy	231/99	38.8 ± 7.0	328.3 ± 78.8
All patients	390/195	39.5 ± 6.4	317.7 ± 106.9
P	0.3	0.474	0.023

## Discussion

Currently, the literature features controversial reports on the relationship between SUA and cerebral infarction. Many studies have found elevated SUA indicative of a poor prognosis (dead or be in care) in vascular event [7], especially elevated SUA increasing the incidence of myocardial infarction and chronic renal failure [15]. Furthermore, large cohort studies have also shown that SUA is an important independent risk factor for cardiovascular mortality and SUA may increase the risk of cardiovascular events? [4,16-19].

Chamorro et al confirmed that patients with good prognosis had higher SUA levels in the acute stage of cerebral infarction classically [12]. In this study, the results showed that young cerebral infarction patients with better prognosis had elevated levels of SUA on admission, and that patients within the lowest quintile of SUA levels suffered from severer dysfunctions compared with those patients within the highest quintile.

Waring et al also demonstrated that SUA reduced exercise-induced oxidative stress in healthy adults. In this study, we also compared SUA levels between age and gender-matched control population, and young patients with cerebral infarction, and found SUA concentrations in healthy controls is higher than in cerebral infarction patients.

In our study, we demonstrated that the worst prognosis was related with the lowest admission SUA levels in cardiogenic cerebral infarction patients. thus, patients with cerebral infarction caused by blockage of small blood vessels had the better prognosis and higher SUA levels on admission, which further illustrates that high SUA level on admission is helpful for functional recovery in young cerebral infarction patients.

In patients with acute stroke, the SUA concentrations decreases significantly over time, and their plasma antioxidant capacity is inversely related to the volume of cerebral infarction and the severity of neurological impairment [20]. Neurological impairment at stroke onset and final infarction size at follow-up are inversely related with the concentration of SUA [12]. Although the SUA level at stroke onset is associated with the presence and duration of atherosclerotic risk factors and the severity of atherosclerotic disease, the odds of good clinical outcome after the ischemic insult increases 12% for each milligram per deciliter increase of SUA [12]. Our study further suggests that SUA confers significant protection as an antioxidant and anti-free radical in young acute cerebral infarction patients, ant that SUA administration may become a new target for neuroprotection in cerebral infarction patients. Waring et al had administrated the UA in healthy control and observed a significant increase in the free-radical scavenging capacity of serum, using two methodologically distinct antioxidant assays. The effect of SUA was substantially greater than that of vitamin C [21].

Why is SUA generally lower in the cerebral infarction patients with poor prognosis? Experimental data can provide some understanding to these findings from several ways: first, ischemic neuronal damage studies have shown that addition of physiological concentrations of UA protects hypothalamic neurons from excitotoxic and metabolic injury in vitro [22]; second, normal SUA concentrations in human plasma was able to almost completely prevent the inactivation of SOD3 by H2O2. Some studies indicate that local SUA concentrations increase during acute ischemic events, which might be a compensatory mechanism that inhibited the increase of free radical activity [23]; thirdly, the elevated SUA concentrations also reduce oxidative stress caused by acute physical exercise, as reflected by plasma 8-iso-PGF2αconcentrations [24]. In our studies, we also found that higher admission platelet concentration resulted in better prognosis. This discovery is very significant, however, because this was not the main aim of the study, we cannot exclude the prognostic influence of these factors. In our future work, we will focus on more meaningful clinical indicators.

With regards to the time of SUA quantification, we made reference to the measurement of Chamorro A et al [12]; we measured the SUA on admission in order to avoid any interference by the intensity of fluid replacement or the heterogeneous administration of medical therapies.

In our analysis, the interfering factors such as creatinine levels, electrolyte levels, and the factor of with or without kidney disease were considered. All selected patients were admitted to hospital within 72 hours in order to remove the impact of time of onset as well as bed rest on the SUA level.

However, two limitations existed which should be taken into account when assessing the results. First, subgroup analysis has been limited by the relatively small sample size, which might also have caused the non-significance of some statistical tests. Second, these patients were not followed up, so that the long-term prognostic significance of early SUA measurements could not be documented, although not included among the goals at the onset of this investigation.

## Conclusion

In this study, we described the relationship between admission SUA and clinical prognosis of young cerebral infarction patients using a validated outcome scale in a large evaluation. Despite some limitations, our study still supports that SUA play a protective role in young cerebral infarction patients through its antioxidant capacity. Further research is needed at both the scientific and clinical levels before routine treatment of SUA can be recommended.

## Competing interests

The authors declare that they have no competing interests.

## Authors' contributions

BZ, QCG, NY and CG participated in study design and patients preparation; BZ and WZZ measurements and analysis; BZ, SXP and JRY carried out data analysis. All authors have read and approved the final manuscript. 

## Pre-publication history

The pre-publication history for this paper can be accessed here:

http://www.biomedcentral.com/1471-2377/10/82/prepub
